# *FLC* and *SVP* Are Key Regulators of Flowering Time in the Biennial/Perennial Species *Noccaea caerulescens*

**DOI:** 10.3389/fpls.2020.582577

**Published:** 2020-11-11

**Authors:** Yanli Wang, Edouard I. Severing, Maarten Koornneef, Mark G. M. Aarts

**Affiliations:** ^1^State Key Laboratory of Protection and Utilization of Subtropical Agriculture Resource, College of Life Sciences, South China Agricultural University, Guangzhou, China; ^2^Laboratory of Genetics, Wageningen University and Research, Wageningen, Netherlands

**Keywords:** flowering time, vernalization, Brassicaceae, perennial, regulation of flowering

## Abstract

The appropriate timing of flowering is crucial for plant reproductive success. Studies of the molecular mechanism of flower induction in the model plant *Arabidopsis thaliana* showed long days and vernalization as major environmental promotive factors. *Noccaea caerulescens* has an obligate vernalization requirement that has not been studied at the molecular genetics level. Here, we characterize the vernalization requirement and response of four geographically diverse biennial/perennial *N. caerulescens* accessions: Ganges (GA), Lellingen (LE), La Calamine (LC), and St. Felix de Pallières (SF). Differences in vernalization responsiveness among accessions suggest that natural variation for this trait exists within *N. caerulescens*. Mutants which fully abolish the vernalization requirement were identified and were shown to contain mutations in the *FLOWERING LOCUS C* (*NcFLC)* and *SHORT VEGETATIVE PHASE* (*NcSVP)* genes, two key floral repressors in this species. At high temperatures, the non-vernalization requiring *flc-1* mutant reverts from flowering to vegetative growth, which is accompanied with a reduced expression of *LFY* and *AP1*. This suggested there is “crosstalk” between vernalization and ambient temperature, which might be a strategy to cope with fluctuations in temperature or adopt a more perennial flowering attitude and thus facilitate a flexible evolutionary response to the changing environment across the species range.

## Introduction

The transition from vegetative to reproductive growth is an important event in the plant’s life cycle and is determined by an interaction between developmental programs and pathways that respond to environmental cues such as day length and temperature (Andrés and Coupland, 2012). In many temperate plant species, including the model plant *Arabidopsis thaliana*, the transition to reproductive growth is accelerated by vernalization. The molecular genetics of the vernalization response has been well studied in *A. thaliana* (Andrés and Coupland, 2012) and many of its components are present in other, especially Brassicaceae, species (Leijten et al., 2018), suggesting that the pathway is conserved although minor differences between species cannot be excluded. In winter-annual *A. thaliana* accessions, the promotion of flowering by vernalization is controlled by the interaction of floral repressors such as FLOWERING LOCUS C (FLC), FRIGIDA (FRI), and SHORT VEGETATIVE PHASE (SVP). FLC is a MADS domain protein that acts as a repressor of flowering (Michaels and Amasino, 1999; Sheldon et al., 1999). Its expression can be activated by FRI, which acts as part of a transcription complex that binds to the *FLC* promoter (Choi et al., 2011). *VERNALIZATION INSENSITIVE 3* (*VIN3*) (Bond et al., 2009; Kim et al., 2010) is the most upstream gene in the vernalization pathway (Kim et al., 2010). The VIN3 protein acts as a partner of a complexes such as LIKE -HETEROCHROMATIN PROTEIN 1 (LHP1) and POLYCOMB REPRESSION COMPLEX 2 (PRC2) to regulate histone methylation at the *FLC* locus, which represses *FLC* transcription (Sung and Amasino, 2004). The decline of *FLC* expression is maintained even when cold-treated plants are returned to warm conditions, thereby relieving the repression of *FT*, a potent activator of flowering and considered the main floral integrator in *A. thaliana* (Turck et al., 2008). In the perennial species *Arabis alpina*, the vernalization requirement and response are controlled by an *FLC* orthologue called *PEP1*. *PEP1* has a complex duplicated gene structure which differs from the simple structure of *PEP1* orthologues in related annual species such as *A. thaliana*. Furthermore, *PEP1* expression is upregulated again when the plants are transferred to warmer temperatures after the vernalization treatment, which implies that meristems that did not become induced, remain vegetative (Wang et al., 2009; Albani et al., 2012). SHORT VEGETATIVE PHASE (SVP) is another negative regulator of the floral transition (Hartmann et al., 2000). It is also a MADS box transcription factor, repressing flowering either in a transcriptional complex with FLC or independent from the latter (Mateos et al., 2015).

*Noccaea caerulescens* (formerly called *Thlaspi caerulescens*), is a diploid (2n = 14), biennial or facultative perennial plant from the *Brassicaceae* family. *N. caerulescens* is an extremophile, adapted to growth on soils with high concentration of Ni, Zn, Pb, or Cd. Next to displaying extreme heavy metal tolerance, it is also a heavy metal accumulator, with genotypes that are able to accumulate Ni, Zn, and Cd to over 1% of their dry weight in shoots (Assunção et al., 2003; Nascimento and Xing, 2006; Broadley et al., 2007; Krämer, 2010). Together with the Zn/Cd hyperaccumulator species *A. halleri*, *N. caerulescens* is among the most prominent plant model systems to study heavy metal hyperaccumulation and associated hypertolerance (Krämer, 2010; Hanikenne and Nouet, 2011; Pollard et al., 2014). *N. caerulescens* is a winter annual, biennial or facultative perennial species, depending on its location and especially water availability during summer. Seeds generally germinate in late summer, early autumn and overwinter as a rosette plant. Most plants will start to flower in early spring, provided rosettes are large enough. In a hot, dry summer, inflorescences will senesce and rosettes will wilt and die after flowering, however, if there is enough water available, either the rosettes remain and will overwinter to flower again next spring, or small secondary rosettes will form at the base of the senesced inflorescence, that will overwinter to flower the next spring. All known accessions will need a vernalization period of 2–3 months (Peer et al., 2003, 2006), which makes molecular genetic studies in this species challenging, as it limits the efficiency of genetic studies (Guan et al., 2008) and breeding efforts to enhance its application in metal phytoremediation. To overcome this disadvantage, two faster-cycling lines, which do not require vernalization, have been generated from the *N. caerulescens* “Ganges” background through fast neutron mutagenesis. The genetic basis of the early flowering phenotype of these lines, and the molecular nature of the mutations involved, is still unclear (Ó Lochlainn et al., 2011).

Associated with the genotypes and the local environments they adapt to, *A. thaliana* accessions show extensive natural variation in their vernalization requirement (Nordborg and Bergelson, 1999). The exact temperatures and length of cold exposure requires that the vernalization response varies among and within this plant species (Kim et al., 2010; Duncan et al., 2015). However, the role of these environmental and additional factors is only partly known in other temperate species. Recently, vernalization requirements and flowering time variations among *N. caerulescens* accessions were reported (Guimarães et al., 2013), but the genetic basis and molecular mechanisms of flowering time regulation has not been studied yet in this species.

A better understanding of the genetic control of flowering time in *N. caerulescens* will help us to understand its morphological and phenotypic behavior as an adaptation to climate conditions. Day lengths of 8 and 12 h did not influence the flowering time when a 4°C cold treatment was applied to induce flowering, indicating that only temperature seems important to induce flowering in *N. caerulescens* (Guimarães et al., 2013). In addition to the natural occurring variation, the identification of early flowering time mutations will also be important for uncovering the key genes involved in the flowering time regulation pathway. The present study investigates the variation of the vernalization requirement and response in four representative *N. caerulescens* accessions from diverse environments. We confirm the essential roles of *FLC* and *SVP* by the identification of mutations in these genes in early non-vernalization requiring plants obtained by forward screening of an EMS-mutagenesis-induced M2 population. Based on our findings, a flowering time regulation model in the biennial/perennial species *N. caerulescens* is proposed, including the effect of high ambient temperatures.

## Materials and Methods

### Plant Materials and Growth Conditions

*N. caerulescens* accessions Lellingen (LE), La Calamine (LC), and Ganges (GA) are obtained by single seed descent propagation as described by Assunção et al. (2003). They originate respectively, from non-metallicolous soil in Wilwerwilz, close to Lellingen in Luxemburg (49°59′1.83′′N, 5°59′39.0′′ E); from calamine soil at the entry to a former Zn mine in La Calamine/Kelmis in Belgium (50°42′38.78′′N, 6°0′37.39.4′′ E); and, most likely, from calamine soil at a former Zn smelter in Les Avinières, close to St. Laurent le Minier in the south of France (43°56′11.2′′N, 3°40′ 17.2′′ E). The accession San Felix de Pallières (SF) has also been collected in the south of France from calamine soil at a former Zn mine (44°2′.40.03′′ N, 3°56′18.05′′ E) and was obtained from Dr. Henk Schat (Free University, Amsterdam, NL). Before this experiment, these accessions have been propagated by self-pollination for at least 4–5 generations (SF) or more than 8 generations (LE, LC, GA) since their collection in the field. Non-vernalization requiring early flowering mutants were identified in an EMS-mutagenized M2 population (approximately 8,000 plants) generated in the inbred SF accession background. Seeds of two early flowering lines (A2 and A7), probably originating from one mutation event, were obtained from Dr. Martin Broadley (University of Nottingham, United Kingdom). These mutants were selected from a fast-neutron mutagenized M2 population, for which the seeds used for mutagenesis were collected from plants growing in the wild in St. Laurent le Minier (Ganges) (Ó Lochlainn et al., 2011) and are further referred to as GA-A2 and GA-A7.

For all plants used in this experiment, the seeds were imbibed at 4°C for 4 days before sowing in pots with a mix of fertilized peat and sand under standard greenhouse conditions (16 h light/8 h dark cycle) set at 23°C and 65% relative humidity. For the flowering time variation study, the plants were vernalized in a cold growth room (at 4°C, 16 h light/8 h dark) for 4, 6, 8, 10, and 12 weeks, after 60 days of vegetative growth in the standard greenhouse conditions, and subsequently transferred back to the same greenhouse. To examine the temperature effect on flowering time, seeds were sown in a growth chamber (16 h light/8 h dark) set at 20°C or 30°C. The rosette leaves and inflorescences distal from open flowers were collected for RNA extraction when the first flower had opened. The inflorescence samples may have contained a few small bracts.

The effect of a cold period on inflorescence number was evaluated by counting the number of inflorescences 30 days after the transfer back to the warm greenhouse. The subsequent inflorescences on the side shoots were not included. Ten plants were scored per accession.

### Gene Expression Analysis

RNA was extracted following a Trizol protocol (Oñate-Sánchez and Vicente-Carbajosa, 2008) and treated with DNA-free DNase (Promega)^1^. RNA quality and quantity were determined using agarose gel electrophoresis and a NanoDrop ND1000 spectrophotometer (NanoDrop Technologies, Wilmington, DE, United States). cDNA was synthesized from 1 μg of total RNA using a cDNA reverse transcription kit (iScript^TM^cDNA Synthesis Kit) following the protocol recommended by the manufacturer.

Gene expression was determined upon quantitative reverse transcription-polymerase chain reaction (qRT-PCR). A 10-fold dilution of the cDNA was used for each reaction by mixing 4 μl cDNA, 1 μl forward and reverse primers (3 μM) and 5 μl SYBR Green quantitative PCR buffer (Bio-Rad, Cat. no.18080-044). The primer sequences of the different genes are presented in Supplementary Table 1. The following PCR protocol was used: denaturation at 95°C for 10 min to activate the DNA polymerase, followed by 45 cycles of denaturation at 95°C for 10 s, annealing at the primer-specific annealing temperature for 30 s and extension at 70°C for 30 s. Following the last cycle, the melting curve was determined in the temperature range 57–95°C. A last step of cooling was performed at 40°C for 10 s. The relative expressions were determined based on ΔCt values. The expression levels were normalized to the house keeping gene tubulin (see Supplementary Table 1). Three to five biological replicates for each treatment or accession were used for the analysis. Before performing the qRT-PCR, the appropriate primer efficiency for each gene was verified.

### Analysis of Candidate Mutant Alleles and Amino Acid Sequences

The coding regions of *NcFLC*, *NcSVP, NcMAF-like-1*, and *NcMAF-like-2* were PCR-amplified from WT and mutant plants by using 2 μl 10 × diluted cDNA (as used for qRT-PCR) as template. PCR was performed using 35 cycles with regular Taq DNA polymerase. The PCR products were gel-purified using NucleoSpin Gel and PCR Clean-up kits^2^ and the purified fragments were sent for DNA sequencing (Eurofins Genome Sequencing Company, Ebersberg, Germany). *NcFLC* and *NcSVP* genomic DNA (gDNA) fragments (∼600 bp) were also amplified and sequenced. The cDNA and gDNA sequences were aligned using Bioedit^3^. The primers were designed using Primer3Plus^4^ based on a preliminary genome sequence of the GA accession (Severing et al., in preparation) or on published cDNA sequences (Lin et al., 2014). The primer sequences can be found in Supplementary Table 1. The amino-acid sequences of FLC from *A. thaliana, Brassica napus*, and *Arabis alpina* and the splice variants of NcFLC and NcSVP were aligned using the Bioedit program.

### Genetic Complementation Analysis of *flc* and *svp* Mutants

The three mutants that carry *flc* mutant alleles were inter-crossed and crossed with putative *svp* mutants and WT. The early-flowering GA-A2 and GA-A7 lines, obtained from the Broadley lab, were also crossed to the *flc-1* and *svp-1* mutants and each other. The F1 plants were grown under control conditions. Flowering time of the F1 plants was determined as the number of days after sowing when the first flower opened. The flowering time of the F2 plants derived from the cross between the early flowering mutants and the SF WT was determined in the same way and the same conditions.

## Results

### Different *N. caerulescens* Accessions Require Different Vernalization Times

**FIGURE 1:**
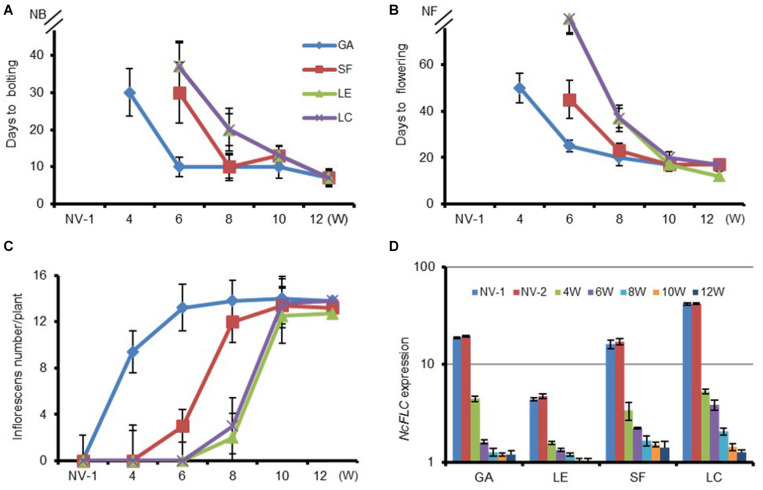
Variation in vernalization response and *NcFLC* expression of *selected N. caerulescens* accessions. **(A)** The transition to flowering as determined by the time to bolting (first flower bud visible) for non-vernalized (NV) plants and for plants vernalized for the indicated number of weeks (W). **(B)** The time to flowering (first petal visible); and **(C)** the number of inflorescences per plant in the same treatments as **(A)**. **(D)** The relative expression of *NcFLC* in rosette leaves upon vernalization for the indicated number of weeks, relative to the expression of *NcFLC* in SF after 12 weeks of vernalization (=1). The expression of *NcTubulin* is used to normalize cDNA concentrations. Values are the mean of three to five plants. Bars show standard errors. NV-1: 2-month-old non-vernalized plants; NV-2: 5-month-old non-vernalized plants. NB, not bolting; NF, not flowering. Accessions are Ganges (GA), St. Felix de Pallières (SF), Lellingen (LE), and La Calamine (LC).

Four *N. caerulescens* accessions, “Ganges” (GA), “St. Felix de Pallières” (SF), “Lellingen” (LE), and “La Calamine” (LC), originating from diverse environments, both in terms of local soil metal concentrations and climate conditions, were examined to determine their vernalization requirements. Sixty days after sowing, the plants were vernalized by keeping them for 4, 6, 8, 10, and 12 weeks in cold (4°C) conditions. The days to bolting (first flower buds visible) and the days to flowering (first petals visible) were determined for each accession (Figures 1A,B). As the length of the cold period increased, the response to the cold treatment varies greatly among the accessions. In general, the prolonged cold treatment accelerated the time to bolting and flowering in all accessions after returning them to warm greenhouse conditions. The largest difference in flowering time among accessions was observed after 4 weeks of cold treatment. Flowering of GA was induced completely with all plants flowering between 50 and 60 days after 4 weeks of vernalization, whereas in SF, LC and LE flowering did not occur until the end of the experiment (180 days after sowing). After a 6-week cold treatment, GA and SF flowered after, respectively, 25 and 45 days after vernalization, whereas LE and LC still did not flower during the whole 180-day period. The number of inflorescences was also higher with a longer cold period. Eight weeks of vernalization shortened the days to bolting in all accessions and increased the inflorescence numbers compared with 6 weeks of vernalization (Figure 1C). For the GA and SF accessions, more inflorescences developed after 8 (vs. 6) weeks of vernalization, whereas in LC and LE, only a few inflorescences developed after this cold treatment. However, the time to flowering was increased in LE and LC. In these accessions some parts of the inflorescences that had bolted did not start flowering at all, indicating that not in all meristems the transition to flowering was completed. After 10 weeks of cold treatment the number of days to bolting and to flowering was reduced to on average, respectively, 10 and 20 days for all accessions, indicating that the vernalization requirement was fully satisfied. After the 10- and 12-week treatments, and transfer to warm conditions, LE flowered the earliest amongst all accessions. It also had stopped flowering, with all siliques being well-developed at 30 days after 10 and 12 weeks of cold, while the other three accessions were still flowering.

### Vernalization Represses *NcFLC* Expression in all Four Accessions

To explore the role of flowering repressors in *N. caerulescens*, we determined the *NcFLC* and *NcSVP* expression levels in the four examined accessions after different vernalization periods. Prolonged cold treatment of the four accessions resulted in a down-regulation of *NcFLC* transcript levels in all of them (Figure 1D), while *NcSVP* transcript levels were not changed with prolonged cold-treatment (data not shown). *NcFLC* expression is already down-regulated in all accessions after 4 weeks of cold treatment and continues decreasing the longer the cold period lasts (Figure 1D). However, the sensitivity to vernalization among accessions does not correlate with the initial expression level of *NcFLC*. Accession LE, with the lowest initial *NcFLC* transcript level among all accessions, flowers only after 10 weeks of cold treatment, and not after 8 weeks, while only minor changes in *NcFLC* expression levels were observed between 8 and 10 weeks of cold treatment in this accession (Figures 1A,B). In the GA, SF and LC accessions, the *NcFLC* transcript threshold to repress flowering seems much higher than in LE, with GA only requiring 4 weeks of vernalization to induce flowering at a higher *NcFLC* expression level than LE.

**FIGURE 2:**
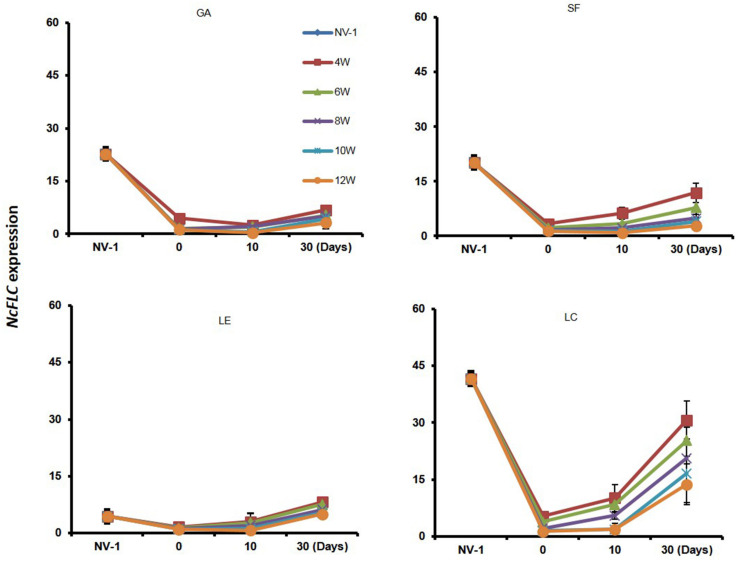
Variation in maintaining repression of *NcFLC* expression upon vernalization. *NcFLC* expression in *N. caerulescens* rosette leaves of accessions Ganges (GA), St. Felix de Pallières (SF), Lellingen (LE), and La Calamine (LC) of plants either non-vernalized (NV-1; 6 months after sowing) or vernalized for 4, 6, 8, 10, or 12 weeks (W) at 4°C after 2 months of growth at 23°C, and subsequently returned to the warm greenhouse. Rosette leaves were collected at 0, 10, and 30 days (Days) following the vernalization treatment. Expression is expressed relative to the expression of *NcFLC* in SF after 12 weeks of vernalization in rosette leaves collected directly after vernalization (=1). The expression of *NcTubulin* is used to normalize cDNA concentrations. Each value represents the mean of three to five plants. Bars show standard errors.

One reason why the four accessions required different weeks of cold for the full acceleration of flowering could be that *NcFLC* expression recovers differently between accessions after the transfer to warmer conditions. Therefore, we also determined the maintenance of *NcFLC* gene expression in the four accessions at 10 and 30 days after the vernalization treatment (Figure 2). After 4–8 weeks of vernalization, the relative *NcFLC* expression indeed increased 10 days after returning to the warm greenhouse in LC, and only marginally in SF and LE, while in GA, *NcFLC* transcript levels stayed constantly low. In all accessions, *NcFLC* expression increased again after 30 days in the warm greenhouse, but the increase was less prominent in GA and LE compared to SF and LC. In general, the *NcFLC* expression remained more repressed the longer the cold period lasted. In GA and SF, however, *NcFLC* transcript levels are relatively more stably repressed than in LE and LC, when the cold treatment lasted up to 10 weeks, at which time flowering is induced in all accessions. This suggests that the recovery of *NcFLC* transcript levels immediately after the transfer to the warm greenhouse, correlates with the absence of flowering after a short period of vernalization.

### Non-vernalization Requiring Early Flowering Mutants in *N. caerulescens*

**FIGURE 3:**
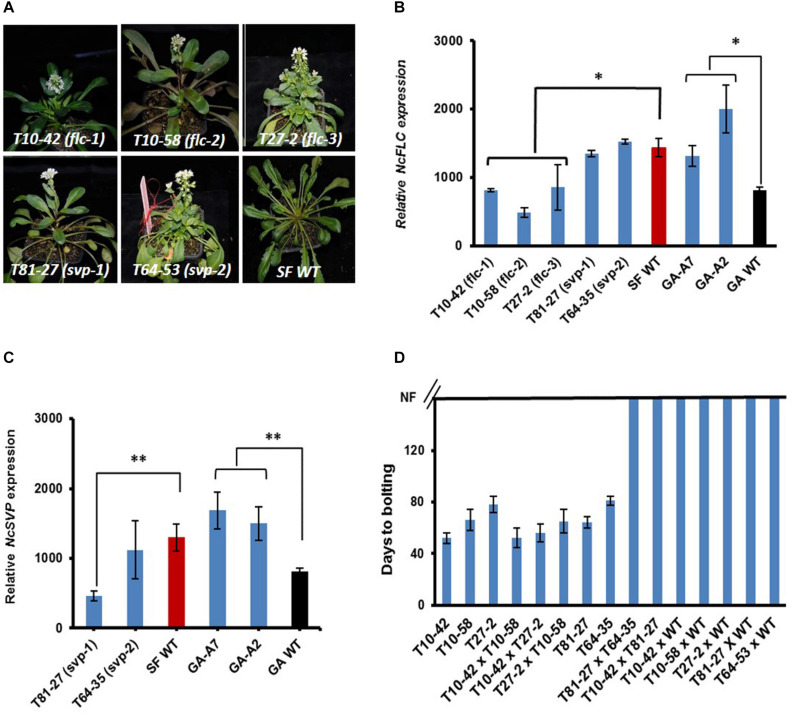
Identification and analysis of early flowering *N. caerulescens* mutants. **(A)** Early flowering mutants (T10-42, T10-58, T81-27, T27-2, T64-35) and their St. Felix de Pallières (SF) wild type (WT) growing without vernalization. The photographs were taken 54, 69, 74, 81, 94, and 94 days after sowing for respectively, T10-42, T10-58, T81-27, T27-2, T64-35, and WT. **(B)** Relative expression of *NcFLC* in rosette leaves of early flowering mutants and WT plants. Both SF and GA WT plants are used. **(C)** Relative expression of *NcSVP* in rosette leaves of early flowering mutants and WT plants. Both SF and GA WT plants are used. **(D)** Days to bolting of self-fertilized and F1 progeny of the early flowering mutants and inter-mutant or mutant-WT crosses growing under non-vernalizing conditions. NF means not flowering. GA-A7 and GA-A2 are the early flowering mutants in Ganges (GA) background. Rosette leaves for expression analysis were collected when the first flower had opened. Expression levels were determined relative to the expression of *NcTubulin* (=1). Each value represents at least three plants ± SE. Asterisks indicate significant differences from WT, *p < 0.05, **p < 0.01, by Student’s *t*-test. Red and black bars in **(B,C)** stand for, respectively, SF WT and GA WT.

To study the molecular mechanisms controlling flowering of *N. caerulescens*, mutants showing an impaired vernalization response were identified upon screening a total of 8,000 M2 seedlings (from 3,500 mutagenized SF plants). During the screen, the day temperature was kept at 20°C and long day conditions (16 h light/8 h dark) were applied. The latter did not induce flowering in the non-mutagenized control SF plants. The screen revealed five early flowering mutants that lacked the obligate vernalization requirement (Figure 3A). The T10-42 mutant is the most extreme early flowering mutant, flowering only 51 days after sowing without vernalization. The T10-58 and T27-2 mutants started flowering, respectively, at 66 and 78 days after sowing. No morphological differences in their inflorescences and flowers were observed between the mutants and their wild-type (WT). We hypothesized that most likely such early flowering mutants would have recessive mutations in floral repressor genes such as *NcFLC* and *NcSVP.* Therefore, we determined the expression of these genes in these early flowering mutants. Three mutants exhibited *NcFLC* transcript levels significantly lower than that of WT suggesting that flowering without vernalization correlated with reduced *NcFLC* transcript level (Figure 3B). Two other mutants exhibited *NcFLC* transcript levels at least as high as those found in the WT. In one of those, mutant T81-27, the *NcSVP* transcript level was significantly lower compared to that in the WT (Figure 3C). For mutant T64-35, as well as for the two lines of the previously published GA mutant (GA-A2 and -A7), the early flowering phenotypes did not relate with significantly reduced expression of *NcFLC* or *NcSVP* (Figures 3B,C).

### Genetic Analysis

To test which mutations are allelic, the non-vernalization requiring mutants were inter-crossed. In addition, the mutants were also back-crossed (BC) to WT. Mutant T64-35 was sterile and no (hybrid) seeds could be obtained. The flowering time of all F1 plants were assessed as the days to bolting after sowing of plants grown in long days in the greenhouse (Figure 3D). All F1 plants derived from crosses between the mutants with low *NcFLC* transcript levels (T10-42, T10-58, and T27-2) flowered without vernalization, indicating that these mutants carry inactive, recessive alleles at the same locus, which means they cannot complement each other (Figure 3D). By contrast, all F1 plants derived from the back-cross with WT (T10-42, T10-58, T27-2, and T81-27) and the F1 hybrids between T81-27 and the three mutants of the first complementation group did not flower in these conditions indicating that T81-27 carries a mutation at a second locus. Analysis of the BC1S1 progeny of the mutant × WT crosses under non-vernalization conditions revealed that all the segregation ratios agreed with a Mendelian 3: 1 ratio of non-flowering: early flowering plants (data not shown), confirming the right crosses were made.

### Splicing-Site Mutations in *NcFLC* and *NcSVP* Result in Early Flowering Mutants

**FIGURE 4:**
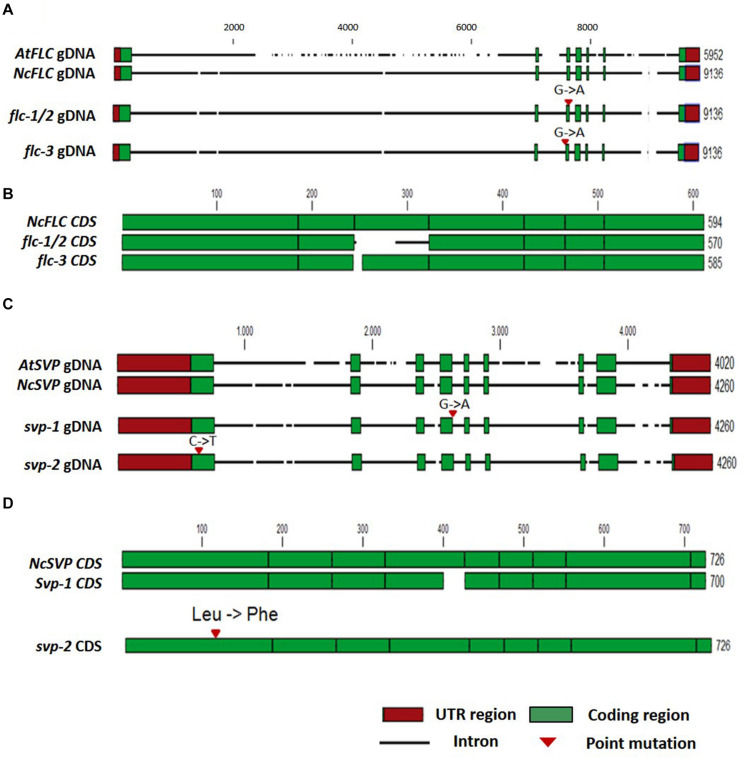
Analysis of the *N. caerulescens FLC* and *SVP* DNA sequences in WT and the *flc* and *svp* mutants. **(A)** Schematic representation of the *Arabidopsis thaliana FLC* genomic DNA (gDNA) sequence (*AtFLC*) compared to the *NcFLC* genomic DNA sequence in *N. caerulescens* WT, *flc-1/flc-2*, and *flc-3* mutants. Numbers indicate total DNA sequence length in base pairs. Intron DNA sequences are indicated with a horizontal black line, with breaks to indicate InDels. 5′ and 3′ untranslated regions (UTR) are indicated with red boxes. Protein coding exons are indicated with green boxes. G to A single base pair mutations are found in all mutants. The mutations in *flc-1* and *flc-2* are identical, suggesting a common mutation event. The mutation in these mutants locates at the 3′ splice junction of exon 3, while the mutation in *flc-3* locates at the 5′ splice junction of exon 3. **(B)** Schematic representation of *NcFLC* coding sequences (CDS) of the *N. caerulescens* WT and *flc* mutants. The G to A mutation in *flc-1/flc-2* causes an in-frame substitution of exon 3 sequence, while the mutation in *flc-3* leads to a 9 bp deletion at the start of exon 3. Exons are indicated in green boxes, the alternative exon 3 in the *flc-1/flc-2* CDS is indicated with a horizontal black line. **(C)** The same as **(A)** for the *SVP* gene. In the *svp-1* mutant, a G to A mutation was found at the 3’ splice junction of exon 4, in the *svp-2* mutant, a C to T mutation was found in exon 1. **(D)** The same as **(B)** for the *NcSVP* CDS. The mutation in *svp-1* causes an in-frame deletion of part of exon 4, while the mutation in *svp-2* leads to a single amino acid change of Leucine to Phenylalanine in exon 1. The numbers at the end of each line indicate the total number of nucleotides, the scale refers to the WT *N. caerulescens* sequence.

Based on the hypothesis that recessive mutations in the floral repressors could result in early flowering, combined with the lower expression levels of *NcFLC* and *NcSVP* (Figures 3B,C) in the early flowering mutants, compared to WT, we cloned and sequenced the *NcFLC* and *NcSVP* cDNAs and genomic DNAs (gDNAs) from all mutants and WT (Figure 4 and Supplementary Figures S1, S2). After alignment of the gDNA and cDNA sequences, G to A point mutations were found, of which two located at different splicing sites of *NcFLC*, one in mutants T10-48 (*flc-1*) and T10-52 (*flc-2*) and one in mutant T27-2 (*flc-3*), while another mutation was found in *NcSVP*, in mutant T81-27 (*svp-1*). Consistent with this, the cDNAs of the *flc* and *svp* variants were alternatively spliced resulting in shorter mRNAs in the mutants than in the WT (Figure 4). The T10-48 and T10-52 mutants (*flc-1* and *flc-2*), which carry the same mutation in the *NcFLC* gene, originated from the same tray of M2 plants (derived from one subset of M1 plants) suggesting that these two mutants are derived from the same mutation event. In the *flc-1* and *flc-2* mutants, the point mutation is located exactly at the exon-intron junction at the 3′ end of the third exon (Figure 4A). This disturbs proper splicing of *FLC* in these mutants substantially. Instead of splicing the 3′ end of exon 2 to the 5′ end of exon 3, the complete exon 3 is skipped, and exon 2 is combined with a new splice acceptor site in intron 3, which adds 38 bp of the end of intron 3 to exon 4. The open reading frame is maintained, but all of exon 3 is replaced with a shorter part of intron 3 in the coding sequence, which has no homology with the original *NcFLC* coding sequence (Figure 4B). In the *flc-3* mutant, the point mutation is located at the intron-exon junction at the 5′ end of the third exon (Figure 4A), also causing aberrant splicing, which results in a 9 bp deletion in the cDNA at the very beginning of the third exon (Figure 4B). Both mutations are predicted to affect the K-domain of the FLC MADS-box protein (Supplementary Figure S1). For *svp-1*, a G to A point mutation was identified at the exon-intron boundary at the 3′ end of the fourth exon (Figure 4C), resulting in aberrant splicing and consequently a 26 bp deletion of the fourth exon in the cDNA (Figure 4D). This deletion causes a frame shift in the open reading frame and the introduction of a premature stop codon just N-terminal to the conserved MADS box domain in the SVP protein (Supplementary Figure S2). To explore whether the early-flowering phenotype in the GA mutant was also due to a mutation in the *NcFLC* or *NcSVP* gene, we sequenced the coding sequences of both genes in the GA mutant. No DNA sequence differences were found in the *NcFLC* and *NcSVP* genes when comparing the GA mutant and its WT type, while these genes are expressed (Figure 3), which shows that this mutant is affected in another gene.

The genetic complementation, together with the expression and sequence analyses, suggest that the identified point mutations explain the loss of *NcFLC* or *NcSVP*, functionality in the *flc-1, flc-2, flc-3*, and *svp-1* mutants, altering their flowering behavior. Sequencing of the *NcFLC* gene in the T64-35 mutant indicated no mutations, but sequencing of the *NcSVP* gene in this mutant revealed a C to T point mutation at position 103 of the cDNA, in the first exon (Figure 4C). This causes a non-synonymous amino acid substitution, altering Leu into Phe, in a conserved region of the predicted protein sequence (Supplementary Figure S2). As this is likely to alter the SVP function in this mutant, we designated this mutant as *svp-2*. Unfortunately, this could not be confirmed by allelism tests because of the complete sterility of this mutant, most likely due to another mutation.

### The Effect of Ambient Temperature on Flowering Time in *N. caerulescens*

So far, the plants used in this study had been selected and grown during winter in a heated greenhouse. When growing the plants in late spring or summer, we noticed that these plants did not flower properly (Supplementary Figure S3). During these periods the day temperature often exceeded 30°C, sometimes even 35°C. This prompted us to examine whether the higher ambient temperatures affect flowering in *N. caerulescens*. WT and *flc-1* mutant plants were grown in climate-controlled growth chamber under long day conditions (16/8 h day/night), with the day temperature set at either 20°C or 30°C and the night temperature at 18°C. The flowering time was determined as the number of days between sowing and bolting of each plant.

**FIGURE 5:**
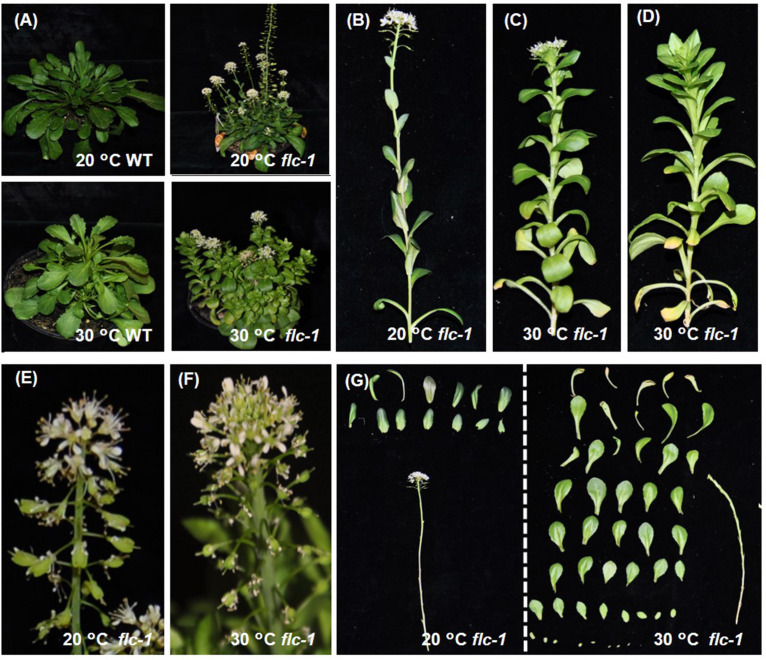
The phenotype of WT and *flc-1* mutant plants at two different day temperatures. **(A)** Flowering plants of WT and the *flc-1* mutant grown for 2 months at either 20 or 30°C. **(B)** The primary inflorescence of the *flc-1* mutant grown at 20°C. **(C)** The primary inflorescence and **(D)** a secondary inflorescence of the *flc-1* mutant grown at 30°C. **(E)** The inflorescence top of the *flc-1* mutant at 20°C and at **(F)** 30°C. **(G)** The leaves on a primary inflorescence of the *flc-1* mutant when grown at 20°C (left) and 30°C (right).

Various morphological differences were observed when comparing *flc-1* plants grown in these two temperatures (Figure 5A). Similar differences were observed for the *svp-1* and the GA-A7 and GA-A2 mutants, which were also grown (Supplementary Figure S4). The *flc-1* plants started bolting 50 days after sowing, in either temperature regime. However, the plants grown at 30°C took 1 week longer until the first flower opened, compared to the plants grown at 20°C. At 20°C, the primary and secondary inflorescences of *flc-1* flowered once 10-13 cauline leaves had developed (Figures 5B,G). At 30°C, at least 30 leaves were formed before the main inflorescence flowered (Figure 5C). Although secondary inflorescences were formed and started to elongate, most of these inflorescences developed more than 50 leaves without any visible flowers (Figure 5D). At 20°C, siliques developed well (Figure 5E) and more than 300 seeds/plant were formed (data not shown). Instead, the flowers that formed on the primary inflorescence at 30°C appeared not to be pollinated or fertilized, with the siliques remaining small and empty (Figure 5F), with no seeds to be obtained. This indicates that the reproductive fitness of the plants is fine at an ambient day temperature of 20°C, but strongly reduced at the higher temperature of 30°C.

### The Effects of Ambient Temperature on Flowering Time and Flower Initiation Gene Expression

**FIGURE 6:**
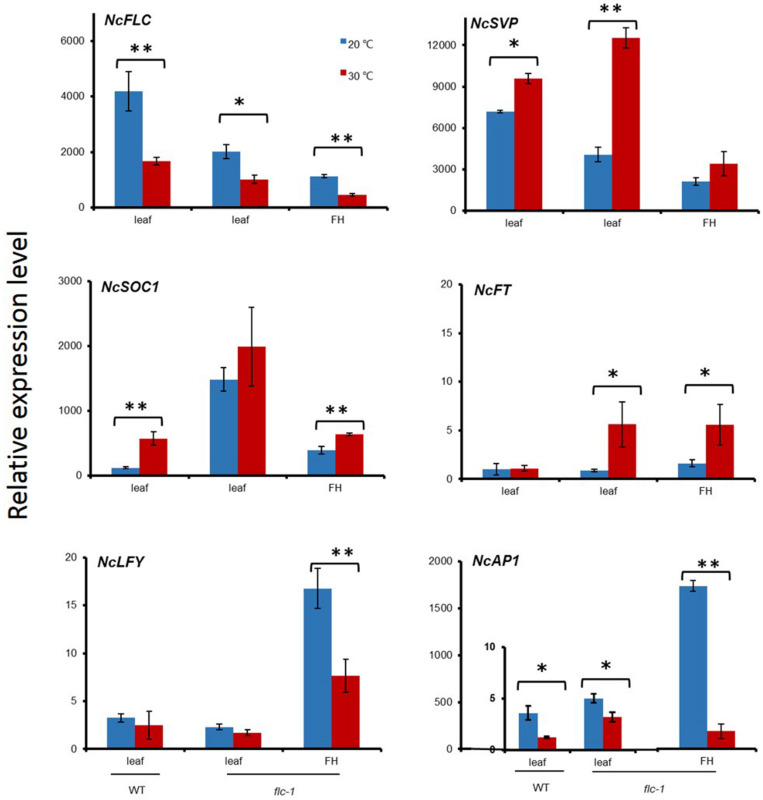
Expression of flowering time regulating *N. caerulescens* genes in different organs and temperatures. The relative expression levels of *NcFLC*, *NcSVP*, *NcFT*, *NcSOC1*, *NcLFY* and *NcAP1* in rosette leaves (leaf) and flowering heads (FH) of St. Felix de Pallières wild-type (WT) and *flc-1* mutant plants grown at 20°C (blue bars) or 30°C (red bars). The expression of *NcTubulin* is used to normalize cDNA concentrations. Expression levels are expressed relative to the expression of *NcFT* in WT (=1). Values indicate the average of at least three plants ± SE. Asterisks indicate significant differences between 20°C and 30°C. *^∗^p* < 0.05, ^∗∗^*p* < 0.01, by Student’s *t*-test.

To further investigate the effects of the day temperature differences on flowering of *N. caerulescens*, the expression of flowering genes was determined in rosette leaves and the inflorescence heads (the top parts of an inflorescence, containing the flower buds and no open flowers) of *flc-1* and WT plants growing at 20°C and 30°C day temperatures (Figure 6). The analysis included the floral repressor genes *NcFLC* and *NcSVP*, the expression of floral integrators *FLOWERING LOCUS T* (*NcFT*) and *SUPPRESSOR OF OVEREXPRESSION OF CONSTANS* (*NcSOC1*) as well as the floral organ identify genes *LEAFY* (*NcLFY)* and *APETALA1 (NcAP1).* Higher *NcFLC* transcript levels were detected in the rosette leaves of WT at 20°C than at 30°C indicating that the temperature influences the expression of *NcFLC*. *NcSVP* expression was up-regulated in rosette leaves at 30°C, compared to 20°C, in both the *flc-1* mutant and the WT, with slightly lower expression observed in the *flc-1* mutant. Both flower promotion genes, *NcFT* and *NcSOC1*, were up-regulated under high temperature, but more up-regulated in leaves of the *flc-1* mutant than in WT under both temperatures. In contrast, the expression levels of the floral organ identify genes *NcLFY* and *NcAP1* were considerably down-regulated in the inflorescence heads at 30°C compared to 20°C. Especially *NcAP1* expression was 14 times higher in the *flc-1* mutant at 20°C than at 30°C.

## Discussion

### Natural Variation for Vernalization Requirement and Response Among *N. caerulescens* Accessions

Plants ensure their reproduction at the most appropriate time and correct stage of development by monitoring their environment and internal signals (Andrés and Coupland, 2012). The accurate regulation of the transition between vegetative and reproductive growth is therefore critical for propagation and survival. The promotion of flowering in response to prolonged exposure to cold temperatures (vernalization) is an adaptation to prevent plants from flowering in the fall, prior to winter, and to enable them to flower in spring. Natural genetic variation in vernalization requirement together with the temperature regimes define when the plant begins to flower and is critical for adaptation to different environments. However, the exact temperatures and length of cold exposure required for the optimal vernalization response vary among species (Kim et al., 2010).

To investigate natural variation for vernalization requirement in *N. caerulescens* we selected four accessions from different environments. Our results provide evidence that vernalization requirement and response among accessions vary within this species (Figure 1). The flowering time differs among *N. caerulescens* accessions depending on the length of the vernalization treatment. Both GA and SF are biennials from a relatively dry region in the south of France, with a short winter (Dubois et al., 2003; Mousset et al., 2016). Consistent with this, a faster vernalization response is predicted to confer a selective advantage to plants that flower earlier thus to avoid early summer droughts and high temperatures that results in poor seed set. LE and LC originate from Luxembourg and Belgium, respectively, and are biennial or facultative perennial accessions, that will have to deal with more severe winters and cooler summers compared to the French accessions. This implies that these accessions should have more opportunities to satisfy the longer vernalization period they require and allowing them to flower later than the accessions from southern France when compared under the same conditions.

### The Identification of Floral Repressors Affected by Vernalization in *N. caerulescens*

Plants that require vernalization to flower, encode repressors that block flowering during summer or autumn, and this block is relieved by reducing expression of the repressor when the plants are exposed to low temperatures (Andrés and Coupland, 2012). The type of floral repressors and the regulatory framework for the vernalization response vary greatly among different species. In *A. thaliana*, expression of the repressor *FLC* drops during vernalization, upon which the transcription of *FT* is induced, which promotes floral initiation (Bastow et al., 2004; Helliwell et al., 2006; Kim et al., 2010). Similarly, SVP acts parallel to FLC to repress flowering in *A. thaliana* (Lee et al., 2007; Mateos et al., 2015). In the preliminary genome sequences of *N. caerulescens* (Severing et al., in prep.) we identified one *NcFLC* and one *NcSVP* orthologue, as well as at least two expressed orthologues of the *MAF* genes (Lin et al., 2014). However, no *NcFLM* and *NcFRI* orthologues were detected at the co-linear sites where they reside in *A. thaliana*. Our finding that early flowering loss-of-function mutants of *NcFLC* and *NcSVP* abolish the vernalization requirement, shows that *NcFLC* and *NcSVP* are the floral repressors in the control of flowering by the vernalization pathway in *N. caerulescens*. The allelic mutants in the GA background (Ó Lochlainn et al., 2011) were not affected in the expression of *NcFLC, NcSVP* or any of the *NcMAF* genes and did not show any mutations in the cDNA sequences of these genes (data not shown), which indicates that there is at least a third locus in *N. caerulescens* required for suppression of flowering in non-vernalizing conditions. Since none of the above-mentioned candidate genes are affected, map-based cloning and genome sequencing will be needed to identify the causal mutation. The observation that single mutants of all three genes result in early flowering indicates that they operate in mutual dependency.

In our analysis, expression of *NcSVP* is not affected by the vernalization treatment (data not shown), though expression is upregulated upon exposure to the higher temperature, while expression of *NcFLC* is downregulated (Figure 6). This suggests that the regulation of *NcSVP* is different from that of *NcFLC*. We did not pursue this any further though. The *svp* mutant flowered slightly later than the *flc* mutants. The Arabidopsis *svp* mutant flowers earlier under short day conditions than under long day conditions, suggesting a photoperiod response of *SVP*, that is not prominent for *FLC* (Albani and Coupland, 2010). Such may also be the case for the *N. caerulescens SVP* gene. Although *N.caerulescens* is typically a vernalization-obligate species, investigating the photoperiod response would enhance the understanding of *NcSVP* functionality.

### *FLC* Expression in *N. caerulescens* Compared to *A. thaliana* and *A. alpina*

The expression pattern of *AtFLC*, and its *A. alpina* orthologue *AaPEP1*, differs between the two species. *AtFLC* remains stably repressed after the plants are transferred to warm temperatures in the winter annual *A. thaliana*, but in the perennial *A. alpina AtPEP1* expression rises again in these conditions, when flowering was induced in meristems present during the vernalization treatment (Wang et al., 2009). The latter has as a consequence that meristems that had not been converted to floral meristems remain vegetative, allowing them to be induced in a new vernalization round in the next season. Analysis of inter- and intraspecies variation demonstrated that the structure of *AaPEP1* is more complex than was found for *AtFLC* (Albani et al., 2012). *A. alpina* contains a tandem duplication of exon 1 of *AaPEP1* which results in two transcriptional start sites and two overlapping transcripts. The organization of *NcFLC* resembles the structure of *AaPEP1* (Supplementary Figure S5), however only one transcript has been detected in *N. caerulescens* (Lin et al., 2014), as in *NcFLC* the sequences of the exon 1 duplications are identical.

Expression analysis of plants after they returned from cold to warm conditions suggested that repression of *NcFLC* is partially stable, especially upon prolonged cold (Figure 2). However, its expression increased gradually upon transfer to the warm greenhouse after a short period of cold, especially in SF. Such increase indicates incomplete vernalization, reactivating *NcFLC* expression to perform the repression on flowering after transfer to a warm greenhouse. A similar phenomenon was also observed in *A. thaliana* accessions requiring very long vernalization periods (Shindo et al., 2006). With the increase of the cold treatment, the *NcFLC* expression is much more stably repressed. Up to 10 weeks of cold treatment, upon which flowering was completely induced in all accessions, the slight increase of *FLC* transcript that was detected was perhaps due to the increased turnover of the mRNA as plant growth accelerated (Shindo et al., 2006). The most likely role of the recovery of the expression level of *NcFLC* after flowering might be related to the maintenance of later formed meristems in a vegetative state, which is essential for perennial species such as *A. alpina* (Wang et al., 2009; Albani et al., 2012). Since both biennial and perennial plants were found in the field for the LE and LC accessions, the perpetual flowering habit might be part of the life style in some *N. caerulescens* accessions. It appears to be rarer for the SF and GA accessions, which may very well be related to the summer conditions at the locations where these accessions are found. These are much warmer and especially drier than those of the more northern accessions, and therefore much less likely to support proper perennial growth of *N. caerulescens*. The partially stable expression of *NcFLC* seems to be intermediate between *A. thaliana* and *A. alpina*, corresponding to the somewhat intermediate life style. Further research, including field observations, will be interesting to fully understand this complex trait.

### The Higher Expression of *FT* and *SOC1* Did Not Promote *LFY* and *AP1* Expression Under High Temperature

During vernalization, leading to a decrease of *NcFLC* expression, *NcFT* and *NcSOC1* expression was upregulated, indicating the repression of *NcFLC* acts through these downstream genes. Both *NcFT* and *NcSOC1* were higher expressed in the non-functional *flc-1 N. caerulescens* mutant compared to its WT indicating that their suppression was eliminated. We observed that plants displayed a different flowering phenotype depending on the growth season (Supplementary Figure S3). When the temperature is higher than 30°C the inflorescences revert to vegetative growth (Figure 5G) and the flowers become sterile. These observations prompted us to check the effect of day-time temperature on the flowering time control in the mutant under two different temperatures. The up-regulation of *NcFT* in the *flc-1* mutant compared to WT suggested that also in *N. caerulescens* the repression of flowering occurs via the repression of the *NcFT* gene (Figure 6). In the *flc-1* mutant, *NcFT* and *NcSOC1* are highly expressed at 30°C. However, the downstream genes *NcLFY* and *NcAP1* were notably lower expressed than at 20°C. We infer that the high expressions of *NcFT* and *NcSOC1* are the direct effect of the high temperature. In *A. thaliana*, alternative spliced isoforms of *AtFLM* and *AtMAF2* were up-regulated under high temperatures. These high-temperature isoforms lost the ability to combine with SVP to repress flowering (Posé et al., 2013; Airoldi et al., 2015). In *N. caerulescens* this scenario is unlikely given that flower initiation has not been observed at elevated temperatures. Alternatively, it was reported that in *A. thaliana* a repression complex formed by LIKE HETEROCHROMATIN 1 (LHP1) and the POLYCOMB REPRESSION COMPLEX 2 (PCR2) is required to maintain repression of *AtFT* (Dilkes et al., 2008). In *N. caerulescens*, the repression of *NcFT* was not maintained under high temperatures indicating that such a complex might also exist in this species. Future research could reveal if maintenance of a similar complex in *N. caerulescens* would be sensitive to high temperature and thus explain the loss of *NcFT* repression. In *A. thaliana*, SOC1, when expressed in the meristem, interacts with AGL24, another MADS box transcription factor, and together they promote the transcription of *LFY*, a meristem identity gene that is involved in the initiation of flower development (Lee et al., 2008). AP1 is also required to initiate and maintain flower meristem identity (Komeda, 2004). The high expression of *NcFT* and *NcSOC1* in the *flc-1* mutant, however, did not induce the expression of *NcLFY* and *NcAP1* at 30°C, suggesting that the promotion of *NcLFY* transcription by NcSOC1 is repressed directly, by the high temperature, or indirectly, by other components involved in this process. Consistent with the expression level of *NcLFY*, the expression level of *NcAP1* in inflorescences at 30°C was much lower than at 20°C. Thus, retarded inflorescence development or reversion from floral to vegetative meristems at 30°C (Figure 5G) is very likely due to the low *NcLFY* and *NcAP1* expression levels. Such reversion might be an advantage in dry and hot, summer, conditions, which would not be favorable for reproduction.

### An Integrated Flowering Regulation Model in *N. caerulescens*

**FIGURE 7:**
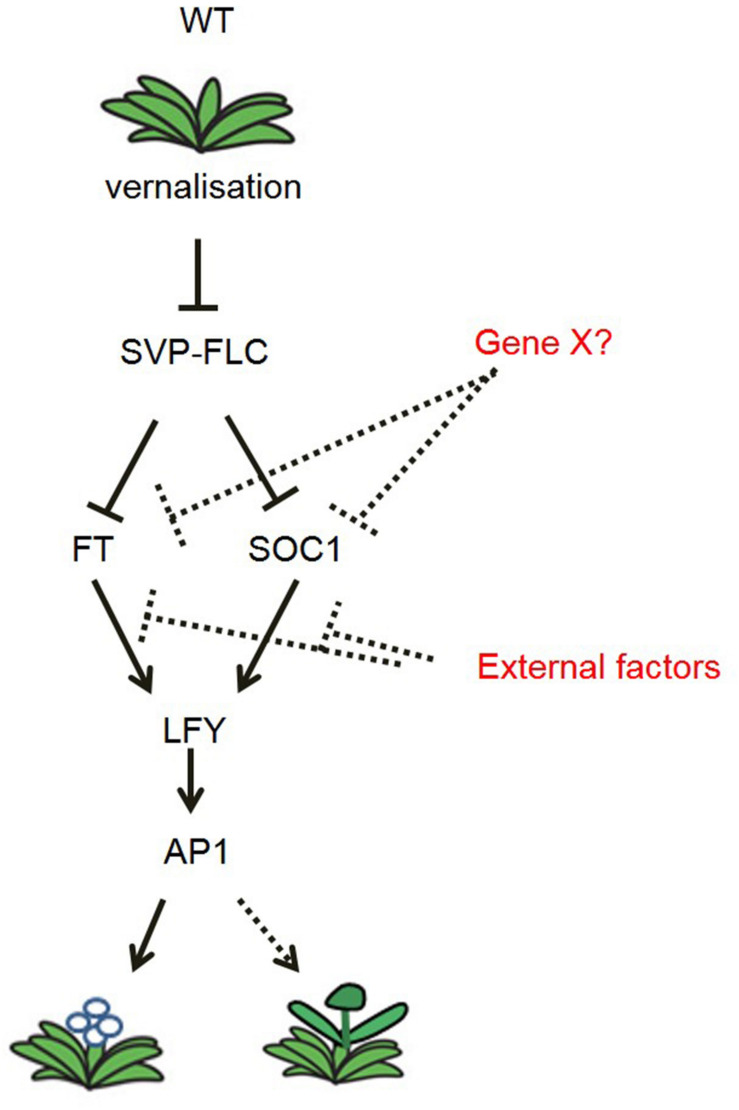
Model of the flowering time regulation pathway in *N. caerulescens*. In general, the vernalization pathway resembles that of *A. thaliana.* In contrast to *A. thaliana*, under high temperature (30°C), *NcFT* and *NcSOC1* are highly expressed, but no longer able to induce the downstream floral identity genes *NcLFY* and *NcAP1*, which delays and disturbs floral initiation at this temperature. Gene X? stands for a third, yet unidentified, floral repressor, which functions parallel to *NcFLC* and *NcSVP.* External factors, e.g., elevated ambient temperature, may suppress the expression of *NcLFY* and *NcAP1* at 30°C. Arrows indicate induction of expression. Bar-ended lines indicate repression of expression. Solid lines indicate confirmed expression effects, while dotted lines indicate interactions still unconfirmed.

The identification of *N. caerulescens* genes that control flowering allows us to compare the molecular pathways controlling seasonal flowering in *N. caerulescens* with those in *A. thaliana*. Based on the transcriptional analysis of key genes in the vernalization pathway, in the non-vernalization requiring mutants, we propose a flowering time regulation model for *N. caerulescens* (Figure 7). In general, this model resembles that of *A. thaliana*. However, the vernalization pathway in *N. caerulescens* is further affected by down-regulation of the floral identity genes *NcLFY* and *NcAP1* under high temperatures, as we expect to happen late in spring or summer. This occurs despite the upregulated expression of floral integrators such as *NcFT* and *NcSOC1*. The temperature sensitivity of the regulating genes *LFY* and *AP1* will be an interesting topic for further study in other biennial or perennial species.

## Data Availability Statement

The datasets presented in this study can be found in online repositories. The names of the repository/repositories and accession number(s) can be found in the article/ Supplementary Material.

## Author Contributions

YW, MK, and MA designed the experiments and wrote the manuscript. YW performed the experiments. ES provided the bioinformatic analysis. All authors contributed to the article and approved the submitted version.

## Conflict of Interest

The authors declare that the research was conducted in the absence of any commercial or financial relationships that could be construed as a potential conflict of interest.

## Footnotes

1 www.promega.com

2 http://www.mn-net.com

3 http://www.mbio.ncsu.edu/bioedit/bioedit.html

4 http://www.bioinformatics.nl/cgi-bin/primer3plus/primer3plus.cgi/
