# Supplementary material for: FLC and SVP Are Key Regulators of Flowering Time in the Biennial/Perennial Species Noccaea caerulescens
Source: Front Plant Sci. 2020 Nov 11;11:582577. doi: 10.3389/fpls.2020.582577 (PMC7686048; doi:10.3389/fpls.2020.582577)
Supplement: Supplementary file 1 [file Data_Sheet_1.PDF]

Supplementary figures

Wang et al.

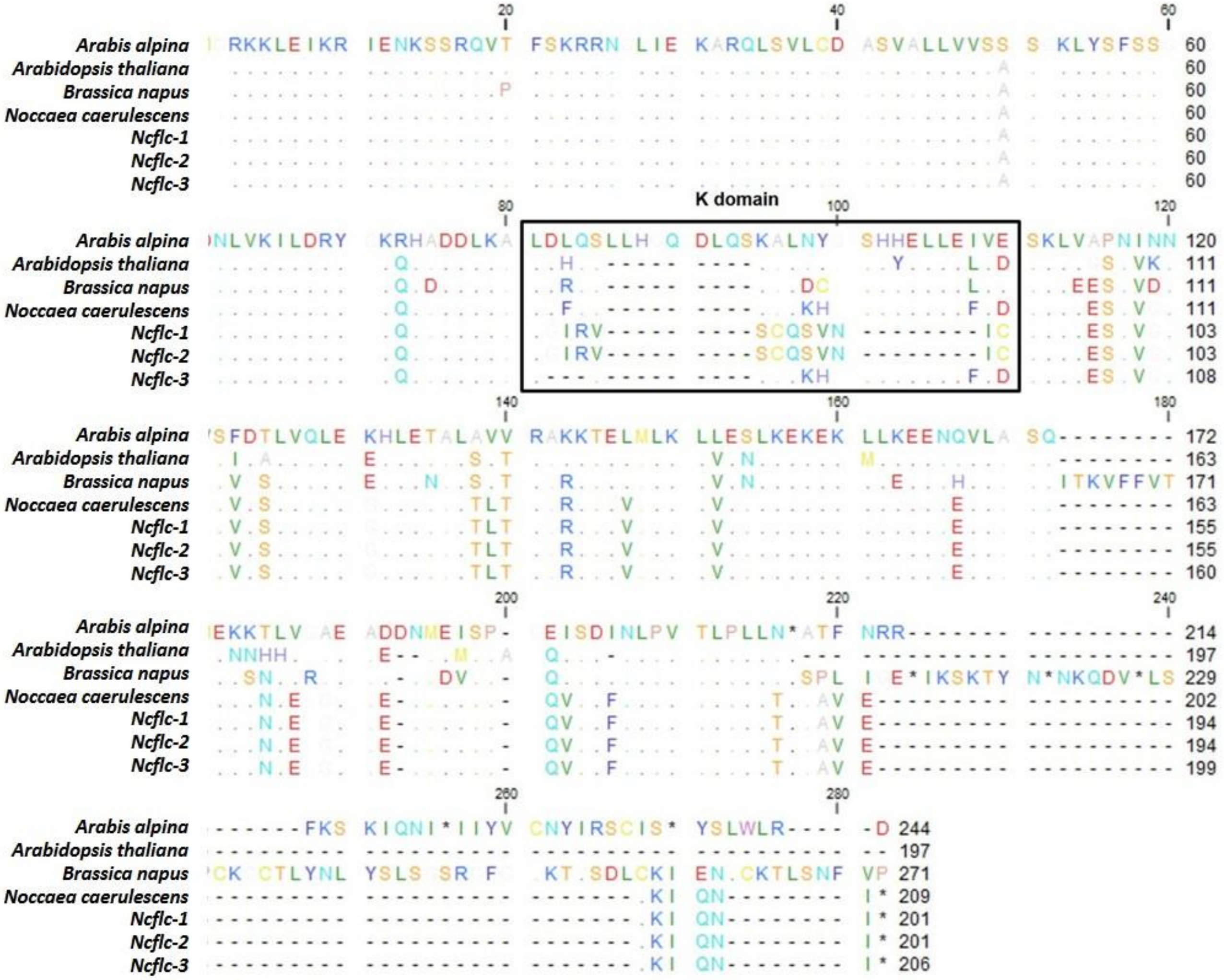

Figure S1. Sequence comparison of FLC MADS box domains in selected Brassicaceae. The predicted amino acid sequences are expressed in single letter code, the conserved MADS-Box K domain is outlined with a black box, dots indicate identical amino acids, dashes indicate deleted amino acids, amino acids different from the *Arabis alpina AaPEP1* reference sequence are indicated. Predicted amino acid sequences of the *Noccaea caerulescens flc* mutants are indicated with “*Ncflc*”.

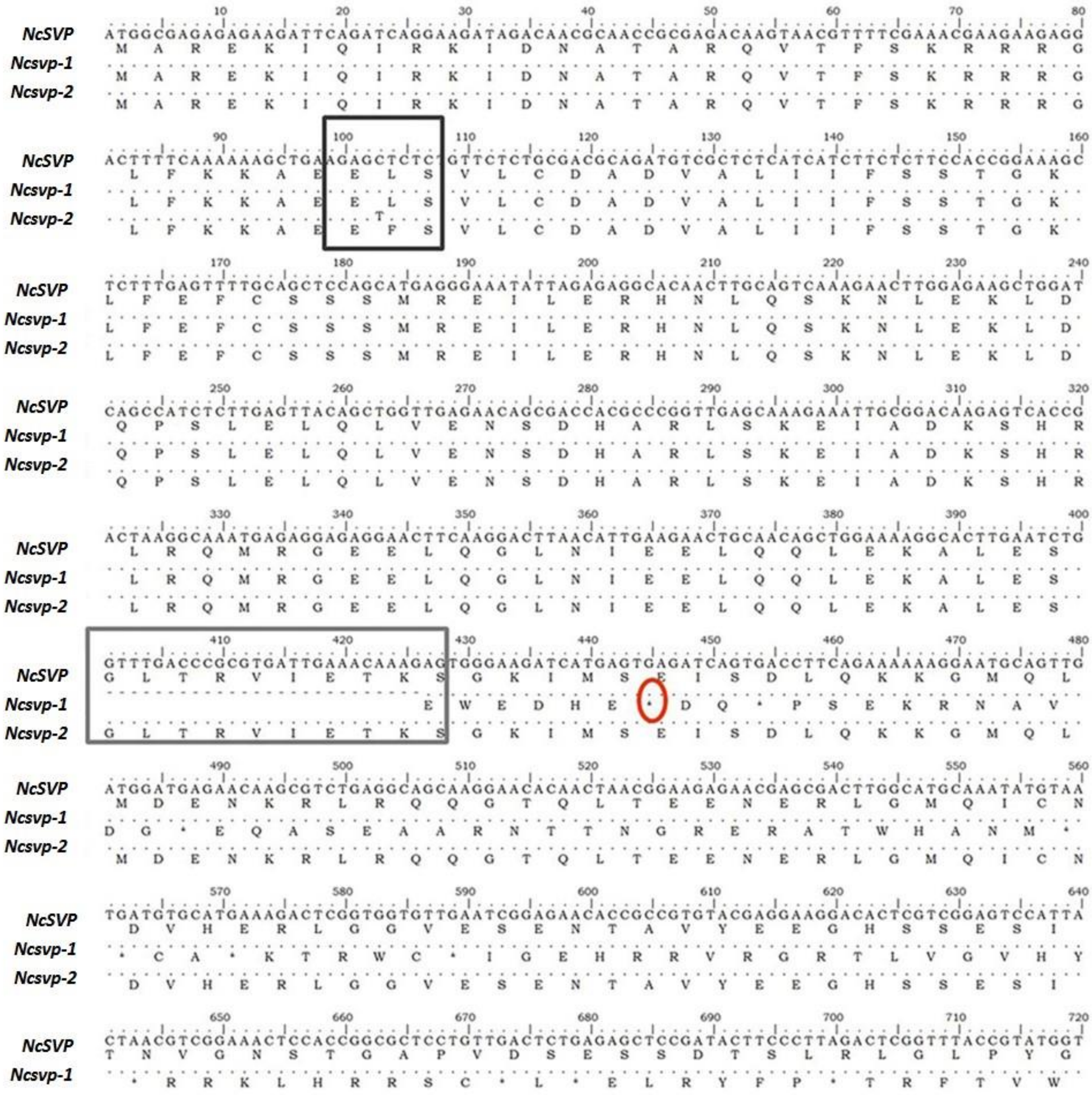

Figure S2. cDNA and predicted amino acid sequences of *NcSVP*. Changes due to mutation in *svp-1* and *spv-2* are indicated. The deletion in the *svp-1* mutant cDNA due to miss splicing is indicated with a grey box, the red circle indicates the first premature stop codon caused by the frame shift due to the deletion. The amino acid substitution from Leu (L) to Phe (F) in *svp-2* is indicated with a black box.

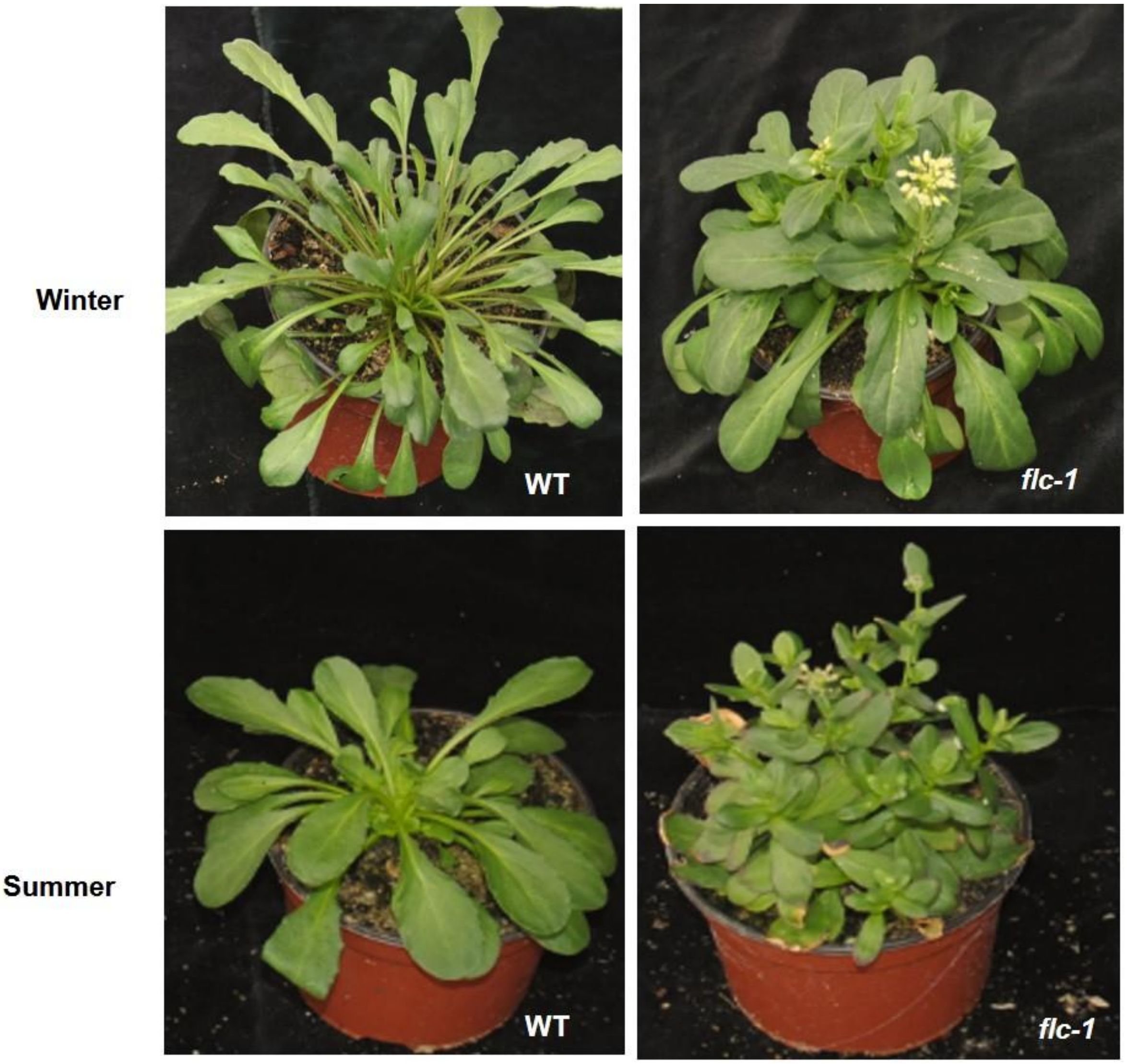

Figure S3. The flowering phenotypes of wild-type (WT) and *flc-1* mutant plants in winter and summer. Plants were grown in a heated greenhouse, in which the *flc-1* mutant flowered and WT did not. In February (Winter), inflorescences developed normally, while in August (Summer), when the greenhouse temperature rose above 30 ℃, inflorescences did not develop properly.

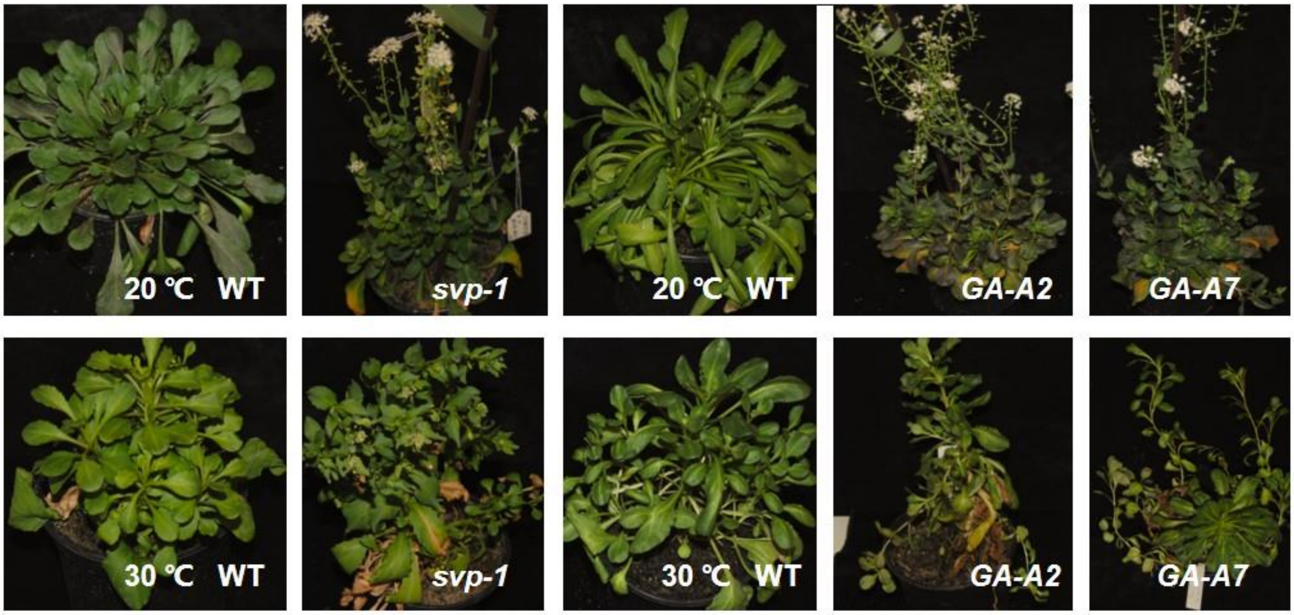

Figure S4. Phenotypes of wild-type (WT) and *svp-1* or *GA-A2/A7* mutant plants at two different day temperatures. Plants are grown for two months at 20 ˚C or 30 ˚C. WT plants do not flower, but early flowering mutants do. At 20 ˚C, the primary and secondary inflorescences of all mutant plants flower properly, but inflorescences are poorly developed at 30 ˚C. WT of *svp-1* is St. Felix de Pallières, WT of GA-A2 and GA-A7 is Ganges.

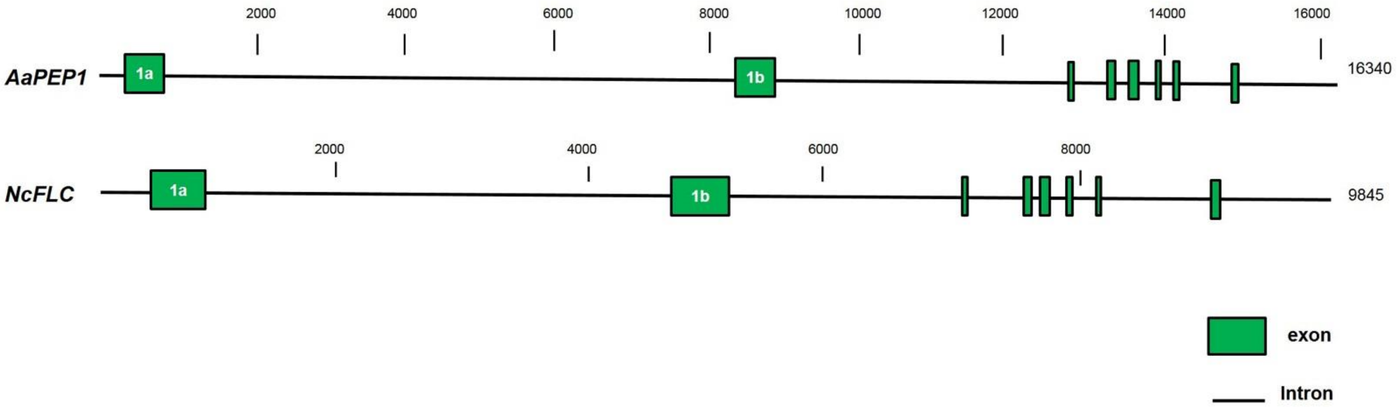

Figure S5. Schematic representation of the *Arabis alpina PEP1* genomic DNA (gDNA) sequence (*AaPEP1*) compared to the *NcFLC* genomic DNA sequence of *N. caerulescens*. In *Arabis alpina*, two transcripts are detected, originating from either copy of exon 1, indicated with 1a and 1b. In *Noccaea caerulescens*, a similar duplication of the first exon sequence is present, but only one transcript is found in the cDNA library presented by Lin et al. (2014), and further confirmed by cDNA sequencing.

Table S1. List of sequences of primers used for qRT-PCR

Gene	Primer	DNA-sequence
	Forward	GTGGAATCAAATGTCGGTAATGTA
NcFLC	Reverse	CTCTAGTCAGAGTGAGGGCTGTCT
	Forward	GCAACCGCGAGACAAGTAAC
NcSVP	Reverse	TGACTGCAAGTTGTGCCTCT
	Forward	ACTTGGTCCCTTACCCGAGAATCC
NcTubulin	Reverse	CATGGAAGCTGGCTCGAAAGC
	Forward	GAGGCATACCAAGGATCGAA
NcSOC1	Reverse	TCTCCCAAGAGTTTGCGTTT
	Forward	CATCGTATCGTGCTGGTATTGT
NcFT	Reverse	CTCACGAGTGTTGAAGTTTTGA
	Forward	ATCTTCCGTTTGGAGCTTCTC
NcLFY	Reverse	GGCGTCTAGAAGATTCCTCCT
	Forward	TTAGGGCACAACAAGAGCAAT
NcAP1	Reverse	CATGTAAGGGTGCTGGATTTG
